# Periodontal Ligament Stem Cells in the Periodontitis Microenvironment Are Sensitive to Static Mechanical Strain

**DOI:** 10.1155/2017/1380851

**Published:** 2017-02-21

**Authors:** Jia Liu, Qiang Li, Shiyu Liu, Jie Gao, Wen Qin, Yang Song, Zuolin Jin

**Affiliations:** ^1^State Key Laboratory of Military Stomatology and National Clinical Research Center for Oral Diseases and Shaanxi Clinical Research Center for Oral Diseases, Department of Orthodontics, School of Stomatology, The Fourth Military Medical University, Xi'an, Shaanxi 710032, China; ^2^State Key Laboratory of Military Stomatology and National Clinical Research Center for Oral Diseases and Shaanxi International Joint Research Center for Oral Diseases, Department of General Dentistry & Emergency, School of Stomatology, The Fourth Military Medical University, Xi'an, Shaanxi 710032, China; ^3^State Key Laboratory of Military Stomatology and National Clinical Research Center for Oral Diseases and Shaanxi International Joint Research Center for Oral Diseases, Department of Oral Histology and Pathology, School of Stomatology, The Fourth Military Medical University, Xi'an, Shaanxi 710032, China; ^4^Department of Stomatology, No. 323 Hospital of PLA, Xi'an, Shaanxi 710054, China

## Abstract

During orthodontic treatment, periodontium remodeling of periodontitis patients under mechanical force was abnormal. We have previously confirmed the function impairment of periodontal ligament stem cells (PDLSCs) in the periodontitis microenvironment which might be involved in this pathological process. However, the response of PDLSCs in periodontitis microenvironment to mechanical force remains unclear. Therefore, in the present study, we introduced a Flexcell tension apparatus and investigated the response of PDLSCs obtained from periodontal tissues of periodontitis patients (PPDLSCs) and of those obtained from healthy periodontal tissues (HPDLSCs) to different magnitudes of static mechanical strain (SMS). PPDLSCs showed increased proliferation, decreased osteogenic activity, activated osteoclastogenesis, and greater secretion of inflammatory cytokines. Different magnitudes of SMS exerted distinct effects on HPDLSCs and PPDLSCs. An SMS of 12% induced optimal effects in HPDLSCs, including the highest proliferation, the best osteogenic ability, the lowest osteoclastogenesis, and the lowest secretion of inflammatory cytokines, while the optimal SMS for PPDLSCs was 8%. Excessive SMS damaged PPDLSCs function, including decreased proliferation, an imbalance between osteogenesis and osteoclastogenesis, and an activated inflammatory response. Our data suggest that PPDLSCs are more sensitive and less tolerant to SMS, and this may explain why mechanical force results in undesirable effects in periodontitis patients.

## 1. Introduction

During orthodontic tooth movement, appropriate orthodontic force can activate biological responses in periodontal tissues [1], including bone formation on the tension side, bone resorption on the compression side, and reattachment of the periodontal ligament (PDL) [2]. In this intricate biological process, the PDL plays a crucial role in maintaining periodontal tissue homeostasis to prevent undesired pathologic conditions [3]. However, currently, orthodontic treatments are no longer confined to adolescents, most of whom have a healthy periodontium. In contrast, an increasing number of adults are attending orthodontic clinics to obtain a charming smile and stomatognathic health, and most of these adults present with mild or severe periodontal disease [4]. Periodontitis with destruction of periodontal tissue and alveolar bone results in increased production of several osteoclastogenic cytokines, such as IL-6, IL-8, IL-1*β*, and TNF-*α* [5, 6]. These cytokines contribute to further periodontal damage. In the absence of inflammatory control, orthodontic treatments could easily lead to rapid loss of periodontal attachment and alveolar bone resorption [7]. Even after the completion of basic periodontal treatment, the regeneration and remodeling capacities of periodontal tissues appear to be decreased in patients with periodontitis [8, 9].

At the cellular level, orthodontic force leads to functional changes in cells in the periodontium. For example, the cell membrane, cytoskeleton, and nuclear protein matrix and genome exhibit functional changes [10]. A considerable amount of evidence has confirmed the presence of adult mesenchymal stem cells (MSCs) in periodontal tissues that maintain tissue homeostasis and regenerative capacity [8]. Periodontal ligament stem cells (PDLSCs) are one of the predominant types of MSCs involved in periodontal tissue regeneration because they not only regenerate cementum- and PDL-like tissues in vivo [11] but also show better organization homology in terms of morphology, structure, and other organizational characteristics [12, 13]. As PDLSCs are load sensitive, studies have shown that mechanical stimulation at the proper strength and frequency promotes the proliferation and osteogenic differentiation of PDLSCs [14]. Moreover, when the PDL is exposed to orthodontic-related mechanical forces, the tissues are reconstructed to balance osteogenesis and osteoclastogenesis. During this process, PDLSCs play a key role in bone formation, while RANKL provides a crucial signal for osteoclast formation [15]. Alveolar bone resorption and periodontal attachment loss occur if the balance between osteogenesis and osteoclastogenesis is disturbed by unsuitable mechanical forces.

Our group previously confirmed that the biological characteristics of PDLSCs are affected by extracellular microenvironment such as inflammation [9, 16] and aging [17, 18]. In the periodontitis microenvironment, the proliferation capability of PDLSCs obtained from patients diagnosed with periodontitis (PPDLSCs) is increased, but the osteogenic and adipogenic potentials are decreased, which induces unfavorable periodontal regeneration. Given that an inflammatory microenvironment can impair stem cell properties, it is reasonable to hypothesize that PPDLSCs respond differently to mechanical forces compared with PDLSCs obtained from healthy periodontal tissues (HPDLSCs), which may lead to elevated osteoclastic activity and alveolar bone resorption in cases of periodontitis. In this study, we evaluated the response of PPDLSCs and HPDLSCs to SMS and investigated the best SMS magnitude for each cell population.

## 2. Materials and Methods

### 2.1. Enrollment of Subjects and Ethics Statement

HPDLSCs for primary cultures were obtained from 10 orthodontic patients (37.9 ± 7.2 years old) undergoing premolar and third molar extractions with healthy periodontal tissue. PPDLSCs for primary cultures were obtained from 8 orthodontic patients (38.9 ± 7.9 years old) who were diagnosed with moderate chronic periodontitis by the same periodontics specialist. The diagnosis was based on the clinical symptoms of positive bleeding on probing, periodontal pocket depth ≤ 6 mm, attachment loss of 3~4 mm, and X-ray images of horizontal alveolar bone resorption by 1/3~1/2 of the root length. We extracted the teeth from the periodontitis patients for orthodontic reasons and harvested the PPDLSCs. To ensure that the biological characteristics of the PDLSCs were not affected by factors other than inflammation, no medicine was used for periodontal treatment. None of the patients in this study had a history of systemic disease, smoking, or special medication. All the samples were collected at the Department of Oral and Maxillofacial Surgery, School of Stomatology, Fourth Military Medical University. All the participants provided written informed consent. The study was carried out in accordance with The Code of Ethics of the World Medical Association (Declaration of Helsinki), and it is approved by the Ethics Committee of the School of Stomatology, Fourth Military Medical University (Xi'an, China).

### 2.2. Cell Culture

The primary cells were cultured, and homogeneous populations of HPDLSCs and PPDLSCs were obtained from single cell-derived colonies by the limiting dilution technique as previously described [9, 11, 16]. These colonies were mixed to obtain multiple colony-derived PDLSCs. Then, stem cells from different individuals were pooled for subsequent use. All the primary cells used in this study were at passage 3.

### 2.3. SMS Loading

For SMS loading, HPDLSCs or PPDLSCs were seeded onto collagen I-coated 6-well Bioflex plates (Flexcell International, Burlington, NC, USA) at a density of 3 × 10^5^ cells/well. Cells achieving 95% confluence were serum starved in *α*MEM for 24 h and then subjected to SMS using a Flexcell Tension Plus system (FX-4000T, Flexcell International, Burlington, NC, USA) capable of producing different strain magnitudes, including 6%, 8%, 10%, 12%, and 14% elongation, at a frequency of 0.1 Hz [19]. The HPDLSCs and PPDLSCs control groups were also seeded onto Bioflex plates but were not subjected to strain. After 12 h of loading or stationary culture, the HPDLSCs or PPDLSCs were used for further experiments.

### 2.4. Flow Cytometric Analysis

Approximately 5 × 10^5^ HPDLSCs or PPDLSCs were incubated with PE-conjugated monoclonal antibodies against human Stro-1, CD146, CD90, CD29, CD31, or CD14 (BD Biosciences, San Jose, CA, USA) for phenotypic identification of PDLSCs. The cells were subjected to flow cytometric analysis using a Beckman Coulter Epics XL instrument (Beckman Coulter, Fullerton, CA, USA) after incubation with specific antibodies as described previously [16].

For cell cycle analysis, HPDLSCs and PPDLSCs (approximately 2 × 10^4^ cells) were cultured in 6-well plates with serum-free *α*MEM for 24 h and exposed to different magnitudes of SMS for 12 h. Then, HPDLSCs or PPDLSCs were prepared and exposed to trypsin/EDTA for 5 min. Cell precipitates were washed twice with 0.01 M PBS and resuspended in 1 mL physiologic saline by repeated vibration to ensure a single cell suspension. Then, 2 mL of cold dehydrated alcohol was mixed quickly with the cell suspension to fix cells at −4°C for 24–48 h. Finally, the cells were washed twice again with PBS, stained with propidium iodide (100 mg/mL; Sigma, St. Louis, MO) at 48C for 30 min, and subjected to flow cytometry (Beckman Coulter). One million cells were counted per sample. The fractions of cells in the G1, S, and G2 phases of the cell cycle were measured, and the proliferation index (PI, the percentage of cells in G2 + S) was determined.

### 2.5. Cell Viability Assays

After SMS loading, HPDLSCs or PPDLSCs were plated at a density of 2 × 10^3^ cells/well in 96-well plates and cultured in basal medium. After 5 days, a 3-(4,5-dimethylthiazol-2-yl)-2,5-diphenyltetrazolium bromide (MTT) assay (Beyotime, China) was performed as previously described [20]. Briefly, 20 *μ*L of MTT agentia was added and the plates were incubated for 4 h at 37°C. Then, 150 *μ*L of dimethyl sulfoxide (DMSO) was added to each well and the plates were incubated for 10 min at room temperature. Subsequently, the absorbance was measured at 490 nm using the plate reader (Sunrise Remote, Tecan).

### 2.6. Real-Time RT-PCR

After SMS loading, total RNA was isolated from HPDLSCs and PPDLSCs (TRIzol; Invitrogen) and converted to cDNA (Super Script First-Strand Synthesis Kit; Invitrogen). Real-time RT-PCR was performed using a QuantiTect SYBR Green PCR Kit (Toyobo, Osaka, Japan) and an Applied Biosystems 7500 Real-Time PCR Detection System. The primers for osteogenic (Runx2, ALP, and OPG) and osteoclastic (RANKL and C-fos) genes are listed in Table 1. Each reaction was performed in triplicate.

### 2.7. Osteogenic and Adipogenic Differentiation Assays

For osteogenesis assays, HPDLSCs and PPDLSCs were exposed to different magnitudes of SMS for 12 h, and after reaching 80% confluence, HPDLSCs and PPDLSCs were cultured in osteogenic medium for 7–21 days and prepared for the differentiation analysis as previously described [21]. Alkaline phosphatase (ALP) staining was performed using the BCIP/NBT Alkaline Phosphatase Color Development Kit (Beyotime, Shanghai, China) at day 7, and ALP activity was determined using an Alkaline Phosphatase (AKP/ALP) Detection Kit (Jiancheng Bioengineering, Nanjing, China) at day 7. Mineralized nodules were stained with Alizarin Red S (pH 4.2) (Kermel, Tianjin, China) for 15 min at room temperature at day 21, and calcium levels were measured quantitatively using a calcium colorimetric assay kit (BioVision, San Francisco, CA, USA). For adipogenic assays, HPDLSCs and PPDLSCs were plated at a density of 5 × 10^4^ cells per well in 6-well plates. After reaching 80% confluence, the HPDLSCs/PPDLSCs were cultured in adipogenic medium (*α*MEM) supplemented with 5% FBS, 0.5 mM methylisobutylxanthine (Sigma, Santa Clara, CA, USA), 0.5 *μ*M hydrocortisone (Sigma, Santa Clara, CA, USA), and 60 *μ*M* indomethacin* (Sigma, Santa Clara, CA, USA) for 7–21 days. For analysis, the adipogenic cultures were fixed in 4% paraformaldehyde for 30 min and stained with fresh Oil Red O solution (Sigma, Santa Clara, CA, USA) for 15 min. To quantify the amount of Oil Red O-stained lipids, the stain was solubilized in isopropanol for 5 minutes at room temperature. The solubilized stain (150 *μ*L) was then transferred to the wells of a 96-well plate, and the absorbance was measured at 520 nm.

### 2.8. ELISA Assays

HPDLSCs and PPDLSCs were exposed to different magnitudes of SMS loading for 12 h and cultured for another 24 h. Then, the concentrations of IL-6, IL-8, IL-1*β*, and TNF-*α* in supernatants from HPDLSCs and PPDLSCs cultures were determined using ELISA kits (Westang, Shanghai, China) according to the manufacturer's instructions.

### 2.9. Statistical Analysis

Statistical analyses were performed using the SPSS 16.0 software package (SPSS Inc., USA). Data acquisition and analyses were performed in a blinded manner. Significant differences in each measure were determined by one-way analysis of variance (ANOVA) and the Bonferroni post hoc test. Additionally, Student's* t*-test was used to analyze the HPDLSCs and PPDLSCs control groups. Statistical significance was set at *P* < 0.05. Data are presented as the mean ± standard deviation. Each experiment was performed three times.

## 3. Results

### 3.1. Culture and Identification of HPDLSCs and PPDLSCs

**Figure 1:**
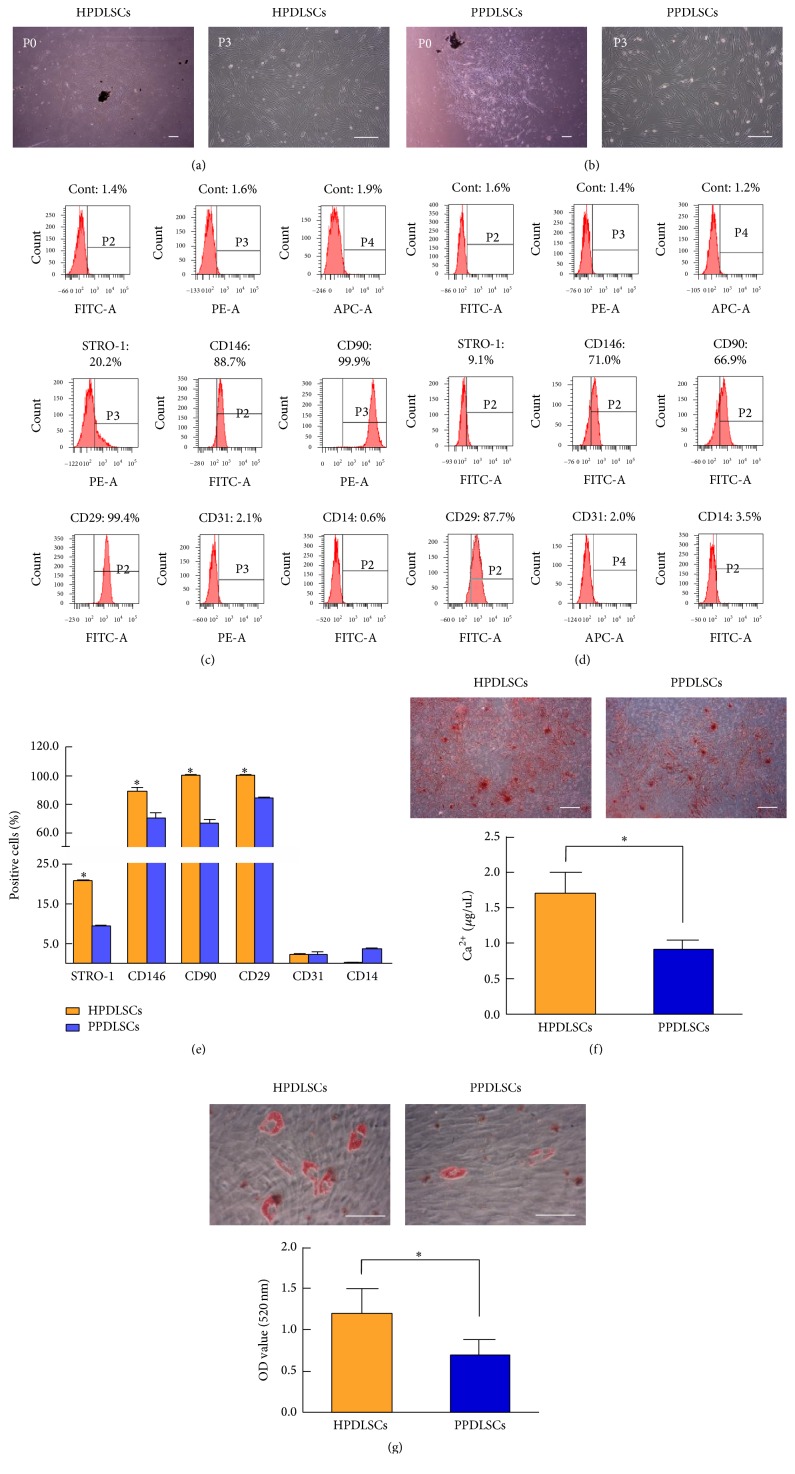
The cell culture and identification of HPDLSCs and PPDLSCs. (a) Morphologies of HPDLSCs observed by microscopy. (b) Morphologies of PPDLSCs observed by microscopy. (c) Mesenchymal stem cell phenotype examination of HPDLSCs by flow cytometric analysis. (d) Mesenchymal stem cell phenotype examination of PPDLSCs by flow cytometric analysis. (e) Quantitative data for mesenchymal stem cell phenotype examination of HPDLSCs and PPDLSCs. (f) Quantitative analysis of mineralized nodule by calcium level analysis. (g). Quantitative analysis of lipids by isopropanol dissolution and absorbance measurement. ^*∗*^*P* < 0.05 versus matched PPDLSCs. Scale bar = 100 *μ*m; *n* = 6 in each group. Each experiment was performed three times. The data are presented as the mean ± standard deviation.

Primary cultures were successfully obtained, and putative HPDLSCs (Figure 1(a)) and PPDLSCs (Figure 1(b)) were isolated. Using a microscope, HPDLSCs and PPDLSCs were observed to grow in an adherent manner with a long spindle shape, and there were no obvious differences between the two cell populations. Both HPDLSCs and PPDLSCs expressed the MSC markers Stro-1, CD146, CD90, and CD29 and were negative for the hematopoietic markers CD31 and CD14 (Figures 1(c) and 1(d)). Compared with PPDLSCs, HPDLSCs showed stronger expression of Stro-1, CD146, CD90, and CD29 (*P* < 0.05). However, there were no significant differences in CD31 and CD14 expression between the two types of PDLSCs (*P* > 0.05) (Figure 1(e)). These data indicated that PPDLSCs from an inflammatory microenvironment more weakly expressed MSC markers compared with HPDLSCs from a healthy microenvironment. Besides, both HPDLSCs and PPDLSCs had the osteogenesis and adipogenesis ability, but due to the impairment of periodontitis, PPDLSCs formed less mineralized nodules and lipid droplets than HPDLSCs (*P* < 0.05) (Figures 1(f) and 1(g)).

### 3.2. Effects of Different Magnitudes of SMS on HPDLSCs and PPDLSCs Proliferation

**Figure 2:**
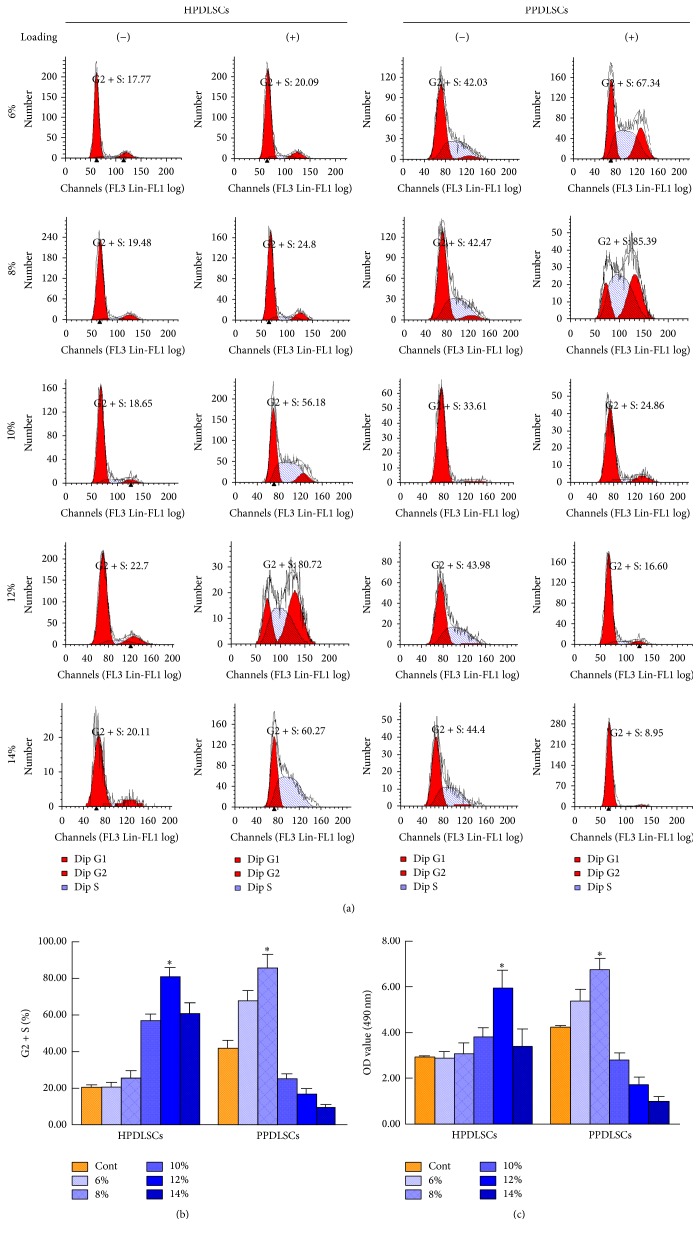
Effects of different magnitudes of SMS on the proliferation of HPDLSCs and PPDLSCs. (a) The percentage of cells in the G2 + S phases was measured by flow cytometry. (b) Quantitative data for the cell cycle analysis. (c) The cell viability was examined by MTT assays. ^*∗*^*P* < 0.05 versus the other five groups of HPDLSCs or PPDLSCs; *n* = 6 in each group. Each experiment was performed three times. The data are presented as the mean ± standard deviation.

To evaluate the effects of SMS on HPDLSCs and PPDLSCs proliferation, these two cell populations were exposed to different magnitudes of SMS, and proliferation was examined by cell cycle analysis and MTT assays. Following inflammatory stimulation, PPDLSCs had a higher PI (Figures 2(a) and 2(b)) and greater cell viability (Figure 2(c)) than HPDLSCs in the control groups of stationary cultures (*P* < 0.05). In the SMS groups, all five magnitudes (6%, 8%, 10%, 12%, and 14% elongation) increased the proliferation of HPDLSCs, but 12% SMS had the best effect based on the PI and MTT index (*P* < 0.05); in PPDLSCs, the best SMS value was 8% (*P* < 0.05), and excessive force decreased both the PI and cell viability (Figures 2(a), 2(b), and 2(c)). These data indicated that SMS differentially influenced the proliferation capacity of HPDLSCs and PPDLSCs. The best SMS value for optimizing proliferation capacity was 12% for HPDLSCs and 8% for PPDLSCs.

### 3.3. Effects of Different Magnitudes of SMS on the Osteogenesis of HPDLSCs and PPDLSCs

**Figure 3:**
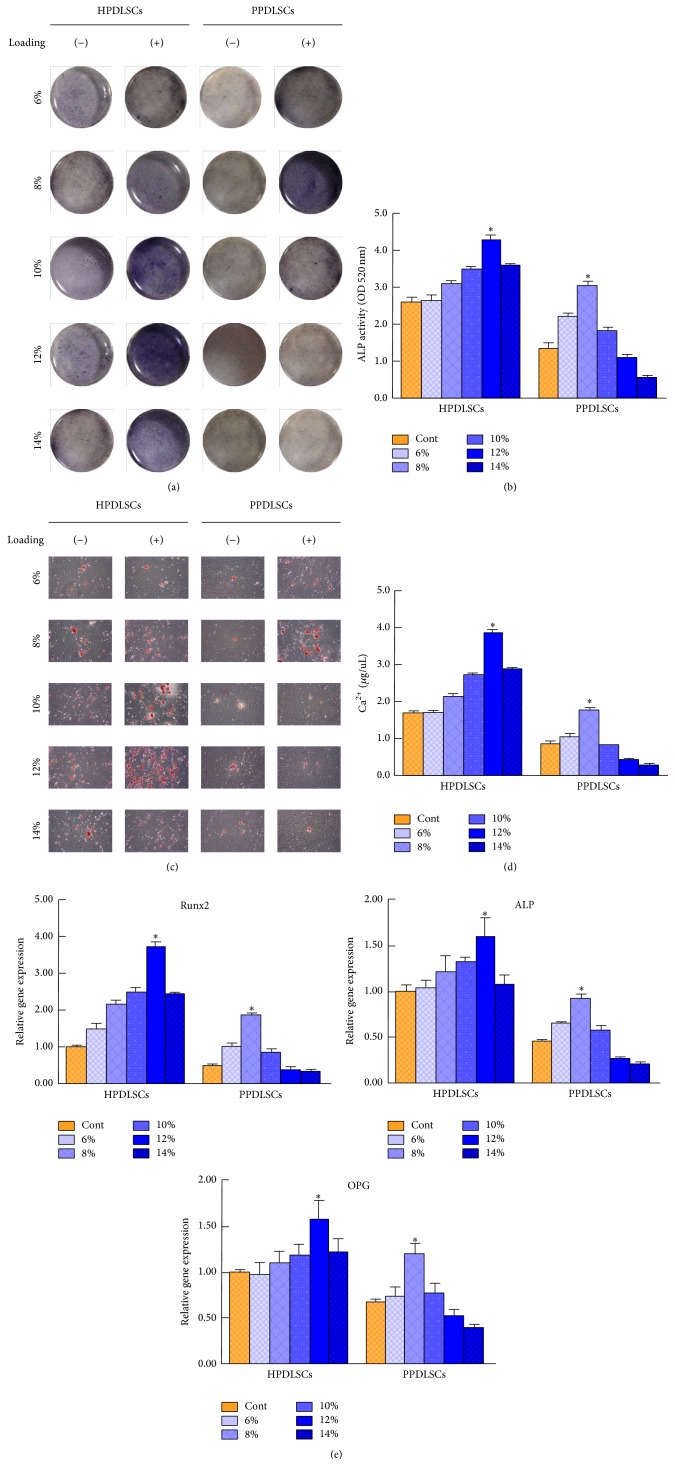
Effects of different magnitudes of SMS on the osteogenesis of HPDLSCs and PPDLSCs. (a) ALP activity was detected by ALP staining. (b) Quantitative analysis of ALP activity. (c) Osteogenic differentiation was determined by Alizarin Red S staining. (d) Quantitative analysis of calcium concentration was performed by calcium level analysis. (e) The expression levels of the osteogenic genes Runx2, ALP, and OPG were measured by real-time PCR. ^*∗*^*P* < 0.05 versus the other five groups of HPDLSCs or PPDLSCs; *n* = 6 in each group. Each experiment was performed three times. The data are presented as the mean ± standard deviation.

To evaluate the effects of different levels of SMS on the osteogenic capacities of HPDLSCs and PPDLSCs, ALP staining, ALP activity assays, Alizarin Red S staining, calcium level analysis, and real-time RT-PCR for osteogenic genes were performed. The results consistently showed that the osteogenic capacity of PPDLSCs was damaged by the inflammatory microenvironment compared with HPDLSCs under conditions with no mechanical stimulation (*P* < 0.05) (Figures 3(a)–3(e)). Exposure to SMS increased ALP activity, mineralized nodule formation, and ALP, Runx2, and OCN gene expression in HPDLSCs in a magnitude-dependent manner until the force reached 12%. However, at 14% SMS, all osteogenic-related indices were decreased (*P* < 0.05) (Figures 3(a)–3(e)). PPDLSCs were more sensitive to SMS than HPDLSCs in terms of osteogenic capacity. The best strain value for PPDLSCs was 8%, and a stronger stimulus had an adverse effect on osteogenic potential, as evidenced by ALP staining, Alizarin Red S staining, and real-time RT-PCR results (*P* < 0.05) (Figures 3(a)–3(e)). These data indicated that the inflammatory microenvironment damaged PPDLSCs function, and the appropriate force level for HPDLSCs was too great for PPDLSCs with regard to osteogenic ability.

### 3.4. Effects of Different Magnitudes of SMS on the Osteoclastogenesis of HPDLSCs and PPDLSCs

**Figure 4:**
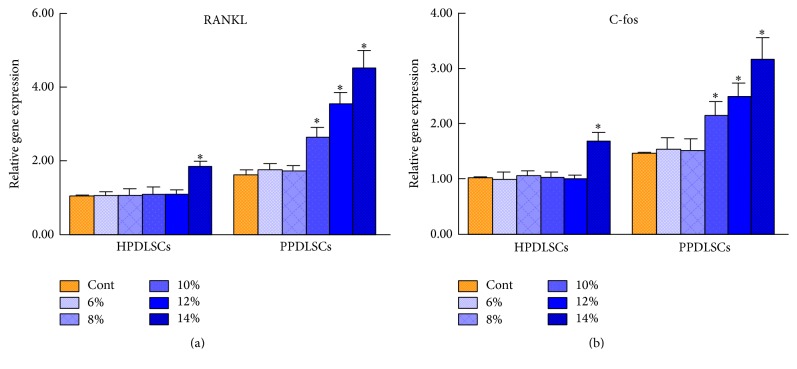
Effects of different magnitudes of SMS on the osteoclastogenesis of HPDLSCs and PPDLSCs. (a) The expression levels of the osteoclastogenic gene RANKL by real-time PCR. (b) The expression levels of the osteoclastogenic gene C-fos by real-time PCR. ^*∗*^*P* < 0.05 versus the control group of HPDLSCs or PPDLSCs; *n* = 6 in each group. Each experiment was performed three times. The data are presented as the mean ± standard deviation.

To investigate the effects of SMS on the osteoclastogenesis of HPDLSCs and PPDLSCs, the osteoclastogenesis-related genes RANKL and C-fos were examined by real-time RT-PCR. The levels of these two genes were higher in PPDLSCs than in HPDLSCs in the control groups without SMS, and this result was attributed to inflammatory stimulation (*P* < 0.05) (Figures 4(a) and 4(b)). In the experimental groups, the mRNA levels of RANKL and C-fos demonstrated no significant differences when the strain was less than 12% in HPDLSCs and less than 8% in PPDLSCs. However, when the strain was higher than 12% in HPDLSCs, the mRNA levels of RANKL and C-fos were obviously increased, and PPDLSCs showed a similar tendency when the strain was higher than 8% (*P* < 0.05) (Figures 4(a) and 4(b)). These data indicated that, in HPDLSCs, an SMS higher than 12% activated the expression of osteoclastogenic genes, while PPDLSCs were more sensitive to mechanical stimulation; an SMS higher than 8% activated osteoclastogenesis in PPDLSCs.

### 3.5. Effects of Different Magnitudes of SMS on the Inflammatory Response of HPDLSCs and PPDLSCs

**Figure 5:**
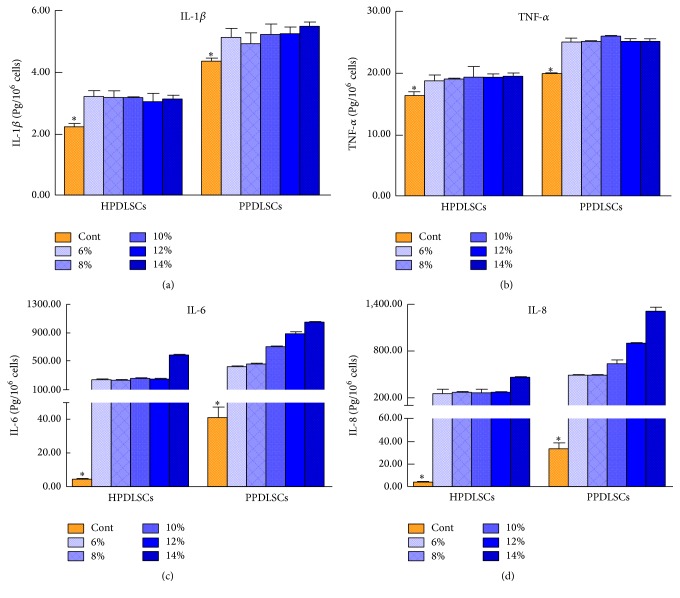
Effects of different magnitudes of SMS on the inflammatory responses in HPDLSCs and PPDLSCs. (a) The protein levels of the inflammatory cytokine IL-1*β* were determined by ELISA. (b) The protein levels of the inflammatory cytokine TNF-*α* were determined by ELISA. (c) The protein levels of the inflammatory cytokine IL-6 were determined by ELISA. (d) The protein levels of the inflammatory cytokine IL-8 were determined by ELISA. ^*∗*^*P* < 0.05 versus the other five SMS loading groups of HPDLSCs or PPDLSCs; *n* = 6 in each group. Each experiment was performed three times. The data are presented as the mean ± standard deviation.

To identify the effects of SMS on the inflammatory response of HPDLSCs and PPDLSCs, the secretion of inflammatory cytokines, including IL-6, IL-8, IL-1*β*, and TNF-*α*, was detected using ELISA assays. PPDLSCs secreted more inflammatory cytokines than HPDLSCs under conditions with no SMS (*P* < 0.05) (Figures 5(a)–5(d)). When SMS was applied, the secretion of IL-1*β* and TNF-*α* by both HPDLSCs and PPDLSCs was slightly increased, but different levels of SMS made almost no difference in each group (*P* > 0.05) (Figures 5(a) and 5(b)). Nevertheless, the levels of IL-6 and IL-8 were significantly upregulated by approximately hundredfold. In identifying the minimal inflammatory response, we found that an SMS less than 12% in HPDLSCs and less than 8% in PPDLSCs induced slight production of IL-6 and IL-8, which was beneficial for tissue regeneration (*P* < 0.05) (Figures 5(c) and 5(d)).

## 4. Discussion

During orthodontic treatment, the transmission of mechanical force to alveolar bone is mediated by the response of the PDL, leading to adaptation of periodontal tissues to the mechanical force [22]. This response primarily depends on cells in the PDL. A study by Theilig et al. [23] showed that cell attachment and expression of the ECM glycoprotein tenascin by human periodontal ligament cells (HPDLCs) were obviously changed after centrifugation. The literature [14] has also documented that low-magnitude and high-frequency mechanical vibration increase ALP activity and OCN expression, ultimately promoting the osteogenic differentiation of PDLSCs in a frequency-dependent manner. However, excessive orthodontic force may be an important cofactor for periodontium damage and the pathogenesis of periodontal diseases. Kim et al. [15] confirmed that periodontal ligament cells (PDLCs) continuously produce RANKL, which provides a crucial signal for osteoclast formation, when subjected to a sustaining orthodontic force. Yamamoto et al. [7] found that PDLCs produced many types of cytokines in response to bacterial stimulation, and in the presence of local inflammation in periodontal tissues, mechanical stress aggravated the inflammatory response and led to alveolar bone resorption.

In our previous study, we found that PDLSCs function was impaired in a periodontitis microenvironment, which may contribute to the deterioration of the regeneration ability of the periodontium [16]. However, the precise mechanisms underlying the different responses of healthy and inflammatory periodontium to mechanical force remained unclear. Furthermore, whether cells derived from tissues in these distinct microenvironments, including HPDLSCs and PPDLSCs, reacted differently to a mechanical stimulus was unknown. The purpose of this study was to evaluate whether SMS exerted different effects on HPDLSCs and PPDLSCs and to determine the optimal SMS value for periodontal remodeling and regeneration by HPDLSCs and PPDLSCs.

We conducted this study by establishing a cell-loading system using a Flexcell FX-4000T device. A numerical analysis of tooth mobility by Natali et al. [24] has demonstrated that maximal PDL strains for translational movements of a human tooth under physiological loading conditions are in the vicinity of 8%–25%, and the value of 12% correlates well with the strain conditions at the midroot. Besides, previous studies have shown that the mechanical strain of 12% along with the frequency of 0.1 Hz was the commonly used force character for periodontal-related cells [19, 25, 26]. Since we assumed that PPDLSCs were more sensitive and thus the optimal value for PPDLSCs should be lower than that for HPDLSCs, we analyzed a range of magnitudes from 6% to 14% with 0.1 Hz frequency to address the purpose of our study. The loading time of mechanical force is important for tissue reaction. Periodontal tissue will remodel when the force is lasting for enough time. When the mechanical strain lasted for 1 h, the viability of PDLCs presented no changes [7]; while the orthodontic force lasted for 12 h, genes and proteins in periodontal tissue began to activate and change [27, 28]. Therefore, we chose 12 h as the loading time in the present study. PPDLSCs were harvested from periodontitis tissues when the periodontitis was in a static phase. Although the PPDLSCs were separated from the inflammatory microenvironment, the functional deficit could not be recovered during ex vivo culture and expansion, according to our current findings and previous studies by our group [16, 29]. Because the periodontitis was chronic in the patients, and the condition was persistent for a long time, we assumed that epigenetic modification could play a critical role in maintaining the functional deficit [30, 31]. It was reported that epigenetics is closely related to inflammation, including in periodontitis [32, 33]. For example, DNA methylation and histone modification have been shown to be critical in the regulation of inflammatory genes [34], and noncoding RNA regulates the expression of target genes at the posttranscriptional level to alter PDLSCs function by epigenetic mechanisms [35]. In our future studies, the specific mechanisms by which epigenetics affect cell function and the response of PPDLSCs to SMS will be considered.

Proliferation is necessary for tissue homeostasis and regeneration by MSCs. In our study, cell cycle and MTT assays were performed to detect the proliferation abilities of HPDLSCs and PPDLSCs. PPDLSCs had a higher proliferation ability than HPDLSCs, which was consistent with previous studies [9, 16]. Moreover, the two populations of PDLSCs responded differently to SMS. The best force value for proliferation potential was 12% for HPDLSCs, while for PPDLSCs, an SMS value higher than 8% suppressed cell viability and decreased the PI. These SMS values indicated that PPDLSCs were more sensitive and less tolerant to SMS than HPDLSCs in terms of proliferation ability.

The process of orthodontic tooth movement should be performed in a manner that balances osteogenesis and osteoclastogenesis, and this balance can be disrupted if the periodontal tissues are in an inflammatory environment [36]. A series of experiments demonstrated that an SMS of 6% to 14% exerted distinct effects on HPDLSCs and PPDLSCs. Specifically, the osteogenic ability of HPDLSCs was upregulated in a magnitude-dependent manner when the cells were exposed to an SMS less than 12%, and the osteoclastogenic genes were barely activated. When the SMS was increased to 14%, the osteogenic ability of HPDLSCs decreased, and the expression of RANKL and C-fos significantly increased, indicating that the balance between osteogenesis and osteoclastogenesis was disrupted. For PPDLSCs, any SMS higher than 8% suppressed osteogenic ability and activated osteoclastogenesis.

Mechanical stimuli can regulate MSCs [37] and PDLSCs [14] functions, including proliferation, differentiation, and cytokine secretion [38]. Excessive inflammatory cytokine secretion can result in periodontal damage; therefore, it is crucial to minimize the production of inflammatory cytokines in the presence of SMS. In our study, the concentrations of IL-1*β* and TNF-*α* in supernatants from both HPDLSCs and PPDLSCs increased slightly under different levels of SMS, but the trend was not significant. Exposure to SMS increased IL-6 and IL-8 secretion to a very high level. We speculated that the mechanical stimulus promoted IL-6 and IL-8 secretion, which might participate in the inflammatory response underlying periodontal remodeling. These findings also suggested that PPDLSCs should receive less SMS than HPDLSCs to minimize inflammatory injury.

## 5. Conclusions

In conclusion, excessive mechanical force and periodontitis-related inflammation led to increased destruction of periodontal tissue, as previously reported [39–42]. Our results demonstrated that HPDLSCs and PPDLSCs responded differently to SMS. Specifically, PPDLSCs were more sensitive and less tolerant to SMS; an SMS of 8% was the best in terms of the regeneration ability of PPDLSCs, while the best magnitude for HPDLSCs was 12%. In the periodontitis microenvironment, excessive mechanical loading can lead to decreased proliferation and osteogenic capacity, the activation of osteoclastogenesis, and increased secretion of inflammatory factors by PPDLSCs, all of which are detrimental for periodontal remodeling in the presence of orthodontic force. These results suggest that an orthodontist should perform light and moderate force to the teeth during orthodontic treatments for patients with periodontitis. However, this study was performed at the cytological level; in future studies, we will determine how to transform the optimal SMS from the cellular level to the tissue level and ascertain the precise mechanisms underlying the differential responses of HPDLSCs and PPDLSCs to SMS.

## Figures and Tables

**Table 1:** Primer sequences.

Gene	Primer sequence
*β*-Actin	Forward 5′-TGG CAC CCA GCA CAA TGA A-3′
	Reverse 5′-CTA AGT CAT AGT CCG CCT AGA AGC A-3′
Runx2	Forward 5′-CCC GTG GCC TTC AAG GT-3′
	Reverse 5′-CGT TAC CCG CCA TGA CAG TA-3′
ALP	Forward 5′-GGA CCA TTC CCA CGT CTT CAC-3′
	Reverse 5′-CCT TGT AGC CAG GCC CAT TG-3′
OPG	Forward 5′-TTGAAATGGCAGTTGATTCCTTT-3′
	Reverse 5′-TATCCTCTTTCTCAGGGTGCTTG-3′
RANKL	Forward 5′-ACCGACATCCCATCTGGTT-3′
	Reverse 5′-GCCATCCTGATTAACTATTAGTT-3′
C-fos	Forward 5′-GACAGCCTTTCCTACTACCATTCC-3′
	Reverse 5′-CGCAAAAGTCCTGTGTGTTGA-3′
